# Risk factors and clinical prediction models for prolonged mechanical ventilation after heart valve surgery

**DOI:** 10.1186/s12872-024-03923-x

**Published:** 2024-05-14

**Authors:** Heng Yang, Leilei Kong, Wangqi Lan, Chen Yuan, Qin Huang, Yanhua Tang

**Affiliations:** 1 Department of Cardiovascular Surgery, The Second Affiliated Hospital, Jiangxi Medical College, Nanchang University, Nanchang, China; 2 The Second Clinical Medical College of Nanchang University, Nanchang, China

**Keywords:** Prolonged mechanical ventilation, Risk factors, Nomogram, Heart valve surgery

## Abstract

**Objectives:**

Prolonged mechanical ventilation (PMV) is a common complication following cardiac surgery linked to unfavorable patient prognosis and increased mortality. This study aimed to search for the factors associated with the occurrence of PMV after valve surgery and to develop a risk prediction model.

**Methods:**

The patient cohort was divided into two groups based on the presence or absence of PMV post-surgery. Comprehensive preoperative and intraoperative clinical data were collected. Univariate and multivariate logistic regression analyses were employed to identify risk factors contributing to the incidence of PMV. Based on the logistic regression results, a clinical nomogram was developed.

**Results:**

The study included 550 patients who underwent valve surgery, among whom 62 (11.27%) developed PMV. Multivariate logistic regression analysis revealed that age (odds ratio [OR] = 1.082, 95% confidence interval [CI] = 1.042–1.125; *P* < 0.000), current smokers (OR = 1.953, 95% CI = 1.007–3.787; *P* = 0.047), left atrial internal diameter index (OR = 1.04, 95% CI = 1.002–1.081; *P* = 0.041), red blood cell count (OR = 0.49, 95% CI = 0.275–0.876; *P* = 0.016), and aortic clamping time (OR = 1.031, 95% CI = 1.005–1.057; *P* < 0.017) independently influenced the occurrence of PMV. A nomogram was constructed based on these factors. In addition, a receiver operating characteristic (ROC) curve was plotted, with an area under the curve (AUC) of 0.782 and an accuracy of 0.884.

**Conclusion:**

Age, current smokers, left atrial diameter index, red blood cell count, and aortic clamping time are independent risk factors for PMV in patients undergoing valve surgery. Furthermore, the nomogram based on these factors demonstrates the potential for predicting the risk of PMV in patients following valve surgery.

## Introduction

Heart valve disease (VHD) comprises a collection of pathological conditions primarily affecting the heart valves and their adjunctive structures [1, 2], resulting in diminished cardiac function, arrhythmias, heart failure, and mortality, thereby imposing a substantial burden on the afflicted individuals’ families and society [3]. The prevalence of heart valve disease in developed nations stands at 2.5%, while individuals aged 75 and above exhibit a prevalence as high as 13.3% [4], a figure expected to rise due to the advancing average life expectancy and the rapid aging of the global population. In contrast, rheumatic heart disease remains the predominant form of VHD in most developing countries, affecting 41 million individuals worldwide [5, 6].

Surgical intervention remains the preferred therapeutic approach for valvular heart disease [7]. Notably, China witnessed over 77,077 heart valve surgeries conducted in 2021 alone. Following cardiac surgery, patients often necessitate mechanical ventilation to facilitate their recovery owing to factors such as general anesthesia, cardiopulmonary bypass, and incomplete restoration of spontaneous breathing [8–10]. While most patients undergoing cardiac surgery are weaned off the ventilator within 12–24 h, a subset of individuals experiences PMV due to various factors [11]. The Society of Thoracic Surgeons (STS) and several studies define PMV as exceeding 24 h [12]. Prolonged mechanical ventilation escalates the risk of respiratory muscle weakness and pulmonary infections [13, 14]. It has also been linked to diminished postoperative quality of life, intensive care unit readmissions, multiple postoperative complications, and increased mortality [15–17]. Therefore, there is an urgent need to identify independent predictors of PMV and establish preventive interventions.

The etiology of prolonged mechanical ventilation after cardiac surgery is multifaceted and has yet to be comprehensively elucidated. Although several investigations have explored the risk factors and prognostic implications of PMV after cardiac surgery, there needs to be more data on the risk factors or predictive models for PMV following valve surgery. This retrospective study aims to identify independent risk factors for PMV following cardiac valve surgery and establish a clinical predictive nomogram model for early identification of PMV.

## Methods

### Study population

The study population consisted of adult patients (age range: 18 years to 75 years) who underwent their initial valve surgery at our hospital between September 2020 and January 2022. Inclusion criteria encompassed patients meeting the specified age range, while patients were excluded if they met any of the following criteria: (1) emergency surgery or prior history of cardiac surgery; (2) presence of severe congenital heart malformations, infective endocarditis, cardiac tumors, or macrovascular disease; (3) coexistence of chronic obstructive pulmonary disease, bronchiectasis, lung infections, or other conditions significantly impacting lung function; (4) presence of severe comorbidities such as liver and kidney failure, autoimmune diseases, malignancies, or infections; (5) significant neurological or psychiatric disorders including delirium, dementia, epilepsy, or a history of schizophrenia; (6) incomplete data. This retrospective study was conducted at a single center and adhered to the principles of the Declaration of Helsinki. The trial received approval from the Ethics Committee of the Second Affiliated Hospital of Nanchang University. The requirement for informed consent was waived due to the observational and retrospective nature of the study, as well as the anonymization of data prior to analysis.

### Anesthesia and cardiopulmonary bypass

Intraoperative rocuronium bromide 0.4 mg/kg/h was continuously pumped to maintain muscle relaxation. Dexmedetomidine and 1% propofol were employed to maintain sedation. The patient was subjected to controlled mechanical ventilation in the Intermittent Positive Pressure Ventilation mode. The respiratory parameters were set: volume control mode, gas flow rate of 2 L/min, tidal volume of 8 ml/kg, inspiratory/expiratory ratio of 1:2, inspiratory pause time of 20%, and the ventilation frequency per minute was adjusted to maintain the end of carbon dioxide partial pressure not higher than 40mmHg. A fraction of inspired oxygen was set at 80%, and 5 cmH_2_O positive end-expiratory pressure(PEEP) was fixed for ventilation. Rocuronium bromide, dexmedetomidine, and 1% propofol were employed to maintain muscular relaxation and sedation. Neuromuscular monitoring was performed by a relaxometry train of four. Sugammadex was administered to reverse the neuromuscular blockage effect of rocuronium. Intermittent sufentanil injections (2 µg/kg) were administered to provide analgesic support, while sevoflurane was intermittently inhaled to ensure the patient remained in general anesthesia.

Patients were administered intravenous heparin sodium at a dose of 3 mg/kg prior to initiation of cardiopulmonary bypass, leading to systemic heparinization. The attainment of an ACT exceeding 480 s served as a pivotal parameter for commencing cardiopulmonary bypass. Employed in this procedure was a JOSPRA apparatus, alongside a membrane oxygenator and single-use cardiopulmonary bypass tubing. Pre-filling was accomplished with Sodium lactate Ringer’s solution, hydroxyethyl starch and sodium chloride injection, and de-whitened suspended red blood cells. Notably, during the final stages of valve surgery and the initial steps of cardiac incision closure, a 1:1 heparin antagonism was executed using protamine sulfate. In instances where the patient’s ACT surpassed their preoperative physiological baseline, an additional dose of 1 mg/kg protamine sulfate was administered. The cessation of cardiopulmonary bypass was executed gradually following the successful resumption of cardiac function. During CPB, mechanical ventilation is stopped. When the heart resumed beating but the CPB had not stopped, the lung resuscitation technique was used first, and three times manual ventilation was used to make the peak airway pressure reach 30 cmH_2_O, which was maintained for 10–15 s, and then half of the initial tidal volume was taken for ventilation. The initial ventilation mode was resumed after the CPB stopped.

### ICU procedures

Subsequent to the conclusion of the surgical procedure, the patient was transitioned to the ICU. The patient was systematically managed in adherence to a standardized protocol. Sustained monitoring encompassed parameters such as non-invasive blood pressure, electrocardiogram, pulse oximetry, central venous pressure, arterial blood pressure, and continuous administration of vasoactive medications to ensure the preservation of stable hemodynamics. In the ICU, postoperative mechanical ventilation was mainly performed in synchronous intermittent command ventilation (SIMV) mode, with an initial setting of tidal volume of 10 ml/kg, oxygen concentration of 60%, PEEP of 5 cmH_2_O, inspiratory and expiratory ratios of 1:1.5-2.0, and a frequency of 14 breaths/minute. Specific ventilator parameters were adjusted according to the patient’s condition. The cessation of sedation is contingent upon a safety evaluation conducted by the ICU team, followed by expeditious extubation. The criteria employed for the postoperative extubation of patients who have undergone heart valve surgery encompass the following facets: (1) Attainment of patient consciousness concomitant with the presence of an intact cough reflex; (2) The assurance of hemodynamic stability, with an absence of arrhythmias, discernible chest tightness, dyspnea, or related symptomatic manifestations; (3) Manifestation of spontaneous respiratory activity, maintenance of partial oxygen pressure exceeding 60 mmHg, FiO2 less than 0.5, and a pH level within the range of 7.35 to 7.45; (4) Maintenance of body temperature above 36.5 °C without the manifestation of chills; (5) The pericardial and mediastinal drainage systems exhibit a restricted flow capacity, characterized by a sustained rate not surpassing 100 milliliters per hour for a duration extending beyond two hours; (6) Preservation of normal peripheral circulation and a urine output rate exceeding 1 mL per kilogram of body weight per hour; (7) Attainment of satisfactory muscle strength in the extremities.

### Data collection

Data regarding the duration of postoperative mechanical ventilation were collected for the study participants. Patients with less than 24 h of postoperative ventilation were classified into the non-postoperative mechanical ventilation (non-PMV) group, while those with more than 24 h of mechanical ventilation were classified into the postoperative mechanical ventilation (PMV) group. Baseline characteristics and clinical data, including demographic information, comorbidities, preoperative variables, and surgical variables, were collected for both groups. If a clinical factor is missing in 20% of patients, this factor will be excluded from the analysis. The missing values of the data will be processed using multiple imputations.

### Definition of essential variables

Postoperative mechanical ventilation exceeding 24 h was classified as PMV. Hypertension was determined by systolic blood pressure ≥ 140 mm Hg, diastolic blood pressure ≥ 90 mm Hg, or antihypertensive medication. Diabetes was defined as fasting blood glucose ≥ 7.0 mmol/L, random blood glucose ≥ 11.1 mmol/L, or the use of diabetic medication. Left atrial diameter index (LADi), left ventricular end-diastolic diameter index (LVDdi), and left ventricular end-systolic diameter index were calculated by dividing left atrial diameter, left ventricular end-diastolic diameter, and left ventricular end-systolic diameter by body surface area (BSA), respectively. Current smokers were defined as patients who had smoked at least 100 cigarettes in their lifetime and were currently smoking. Current alcohol drinkers consumed alcohol more than once per week during the previous six months.

### Statistical analysis

Statistical analyses were conducted utilizing SPSS Statistics 26.0 and R software. Continuous data underwent normality assessment via the Kolmogorov-Smirnov test. Normally distributed data were presented as mean ± standard deviation and analyzed using t-tests. Non-normally distributed data were expressed as median (first quartile - third quartile) and compared between groups using the Mann-Whitney U test. Categorical data were presented as counts and percentages (%) and compared using the chi-square or Fisher’s exact test. Rank data were analyzed using the rank sum test, and univariate analysis was performed to identify potential factors influencing PMV. Variables exhibiting statistically significant differences (*p* < 0.05) were further incorporated into multivariate logistic regression analysis. A nomogram was constructed based on the outcomes of the multivariate logistic regression analysis. The cohort was randomly divided into training and validation cohorts in a 7:3 ratio. The discriminative power of the nomogram was evaluated using the Harrell consistency index (C index) or the area under the receiver operating characteristic curve. Internal validation was conducted employing the bootstrap method with 1000 replications. Calibration curves assessed the concordance between the predicted and observed probabilities derived from the nomogram. Additionally, decision curve analysis (DCA) was conducted to assess the clinical usefulness of the column line plot by measuring the standardized net benefit at various risk thresholds.

## Results

### Patient characteristics

**Fig. 1:**
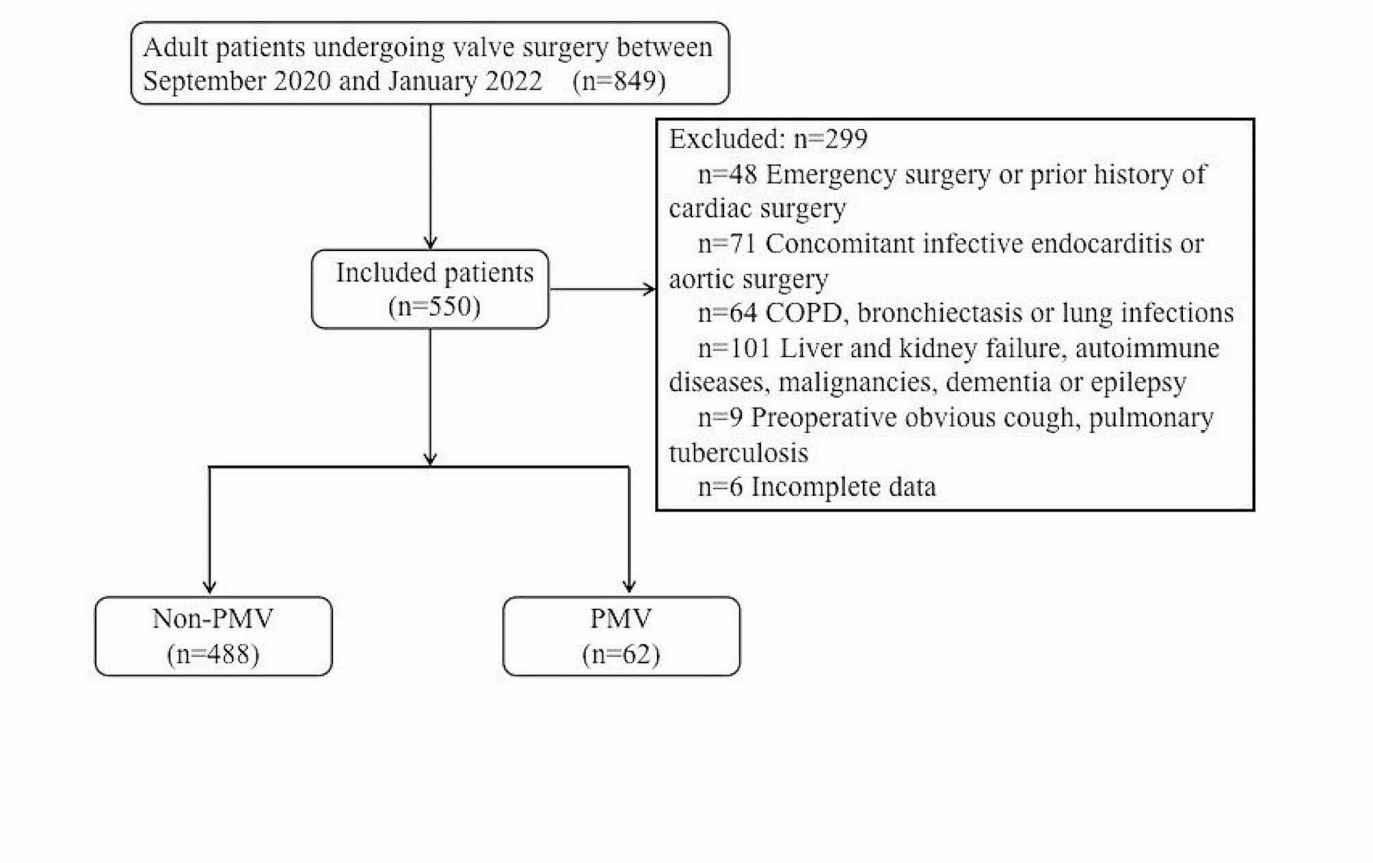
Flow diagram of patient selection

This single-center retrospective study screened 849 patients undergoing valve surgery between September 2020 and January 2022; 299 patients were excluded, and 550 were included in the final analysis (Fig. 1). The median duration of mechanical ventilation after surgery was 19.5 h. Among them, 62 patients (11.27%) developed postoperative PMV, with a median mechanical ventilation time of 26.75 h in the PMV group. The remaining 488 patients did not experience PMV postoperatively, with a median mechanical ventilation time of 19 h in the non-PMV group. The median age of patients in the PMV group was 61.5 years, compared to 54 years in the non-PMV group. In the PMV group, 61.3% of patients were male, compared to 50.4% in the non-PMV group. Hypertension was present in 25.8% of the PMV group, coronary atherosclerosis in 9.7%, diabetes mellitus in 8.1%, and a history of cerebral infarction in 14.5%. Conversely, in the non-PMV group, 20.5% had hypertension, 7% had coronary atherosclerosis, 5.1% had diabetes mellitus, and 7.8% had a history of cerebral infarction. The two groups’ baseline characteristics, preoperative data, and procedure-related data are presented in Tables 1 and 2, and 3, respectively.

**Table 1:** Baseline characteristics of included patients with or without PMV

Characteristics	Non-PMV(*n* = 488)	PMV(*n* = 62)	χ^2^/Z	*P*-value
**Sex, n (%)**			2.608	0.106
Male	246 (50.4)	38 (61.3)		
Female	242 (49.6)	24 (38.7)		
**Age, y**	54 (47–61)	61.5 (56–66)	-5.317	0.000
**BMI, kg /m** ^**2**^	22.52 (20.31–24.65)	21.52 (18.22–24.51)	-2.045	0.041
**Blood pressure, mmHg**				
SBP	120 (110–133)	118 (109.5–132)	-0.549	0.583
DBP	72 (65–80)	70.5 (64–78)	-1.346	0.178
**HR, BPM**	79.5 (71–89)	76.5 (68–84)	-2.095	0.036
**Cardiac function (NYHA), n (%)**			-0.102	0.918
II	100 (20.5)	7 (11.3)		
III	380 (77.9)	54 (87.1)		
IV	8 (1.6)	1 (1.6)		
**Main diagnosis, n (%)**			4.278	0.644
Mitral stenosis	25(5.12)	4(6.45)		
Mitral insufficiency	81(16.6)	8(12.9)		
Mitral stenosis with insufficiency	121(24.8)	13(20.97)		
Aortic valve stenosis	35(7.17)	4(6.45)		
Aortic valve insufficiency	40(8.2)	5(8.07)		
Aortic valve stenosis with insufficiency	81(16.6)	8(12.9)		
Combined aortic and mitral valve disease	105(21.52)	20(32.26)		
**History of cerebral infarction, n (%)**	38 (7.8)	9 (14.5)	3.187	0.074
**Carotid atherosclerosis, n(%)**	44 (9.0)	6 (9.6)	0.029	0.865
**Hypertension, n (%)**	100 (20.5)	16 (25.8)	0.934	0.334
**Coronary arteriosclerosis, n (%)**	34 (7.0)	6 (9.7)	0.265	0.607a
**Diabetes, n (%)**	25 (5.1)	5 (8.1)	0.441	0.507a
**Current_smoker, n (%)**	90 (18.4)	18 (29.0)	3.909	0.048
**Current_drinker, n (%)**	28 (5.7)	5 (8.1)	0.528	0.467

Data are presented as n (%) or median (interquartile range). BMI, body mass index; SBP, Systolic blood pressure; DBP, diastolic blood pressure; HR: Heart Rate; BPM, Beat Per Minute; NYHA, New York Heart Association

**Table 2:** Preoperative factors of included patients with or without PMV

Variables	Non-PMV(*n* = 488)	PMV(*n* = 62)	χ2/Z	*P*-value
**AAD, mm**	31 (29-35.75)	33 (18–25)	-1.904	0.057
**LADi, mm/m** ^**2**^	27.9 (23.52–32.43)	29.89 (25.91–36.07)	-2.497	0.013
**LVDsi, mm/m** ^**2**^	21.62 (18.89–25.42)	22.5 (18.78–26.32)	-0.564	0.573
**LVDdi, mm/m** ^**2**^	32.78 (29.38–36.40)	34.02 (28.97–40.64)	-0.915	0.36
**LVEF, %**	61 (55–66)	61.5 (53–66)	-0.779	0.436
**NT-proBNP, ng/L**	304.7 (103.61-921.38)	583.83 (236-1345.59)	-2.909	0.004
**CRP, mg/L**	2.6 (1.59–6.27)	2.26 (1.56–4.27)	-0.528	0.598
**ESR, mm/h**	15 (7-28.11)	19 (8–15, 18–37)	-1.697	0.09
**Fibrinogen, g/L**	2.48 (2.13–2.98)	2.45 (2.13–3.03)	-0.343	0.731
**Blood Glucose, mmol/L**	4.88 (4.43–5.56)	4.83 (4.41–5.58)	-0.238	0.812
**HbA1c, %**	5.51 (5.23–5.9)	5.7 (5.3-6.0)	-1.536	0.126
**STB, µmol/L**	14.7 (10.7-19.36)	12.96 (10.41–19.78)	-0.688	0.491
**Cr, µmol/L**	72.62 (61.74–86.53)	74.28 (63.75–90.76)	-0.875	0.381
**BUN, mmol/L**	5.99 (4.68–7.33)	6.17 (5.11–7.97)	-1.336	0.182
**UA, µmol/L**	362.96 (300.2-455.89)	373.77 (306.41-438.79)	-0.155	0.877
**K**^**+**^, **potassium ion, mmol/L**	3.86 (3.64–4.15)	3.89 (3.63–4.17)	-0.477	0.655
**Ca**^**2+**^, **calcium ion, mmol/L**	2.32 (2.24–2.39)	2.32 (2.24–2.4)	-0.295	0.768
**WBC count, ×10** ^**9**^ **/L**	5.71 (4.74–6.89)	4.98 (4.4–6.5)	-2.388	0.019
**RBC count, ×10** ^**12**^ **/L**	4.38 (4.04–4.73)	4.1 (3.77–4.44)	-3.863	0.000
**Platelet count, ×10** ^**9**^ **/L**	191 (154.25–220)	162 (142.5-202.8)	-2.298	0.022
**Neutrophil count, ×10** ^**9**^ **/L**	3.45 (2.66–4.41)	3.04 (2.44–4.56)	-1.394	0.163
**Lymphocyte counts, ×10** ^**9**^ **/L**	1.5 (1.2-2)	1.55 (1.1–1.9)	-0.34	0.734
**Monocyte count, ×10** ^**9**^ **/L**	0.37 (0.26–0.5)	0.4 (0.26–0.53)	-0.883	0.377

Data are presented as n (%) or median (interquartile range). AAD, Maximal ascending aortic diameter; LADi, Left atrial diameter index; LVDsi, Left ventricular end-systolic diameter index; LVDdi, Left ventricular end-diastolic diameter index; LVEF, Left ventricular ejection fraction; NT-proBNP, N-terminal pro-brain natriuretic peptide; CRP, C-reactive protein; ESR, Erythrocyte sedimentation rate; HbA1c, Glycated hemoglobin A1c; STB, Serum total bilirubin; Cr, Creatinine; BUN, Blood urea nitrogen; UA, Uric acid; WBC, white blood cell; RBC, Red blood cell

**Table 3:** Intraoperative factors of included patients with or without LMV

Variables	Non-PMV(*n* = 488)	PMV(*n* = 62)	χ2/Z	*P*-value
**Aortic cross-clamp time, min**	67 (53–86)	82 (61.5–101)	-3.102	0.002
**CPB time, min**	97 (79.25–119)	112.5 (82.5-136.5)	-2.699	0.007
**Intraoperative blood loss, ml**	600 (500–800)	600 (500–800)	-1.606	0.108
**Intraoperative blood transfusion, n (%)**	49 (8)	12 (19.4)	4.839	0.028
**Surgery type I**			1.151	0.283
**Valve Replacement, n (%)**	435 (89.1)	58 (93.6)		
**Valve Repair, n (%)**	53 (10.9)	4 (6.5)		
**Surgery type II**			2.521	0.284
**Aortic valve surgery, n (%)**	157 (32.2)	16 (25.8)		
**Mitral valve surgery, n (%)**	230 (47.1)	28 (45.2)		
**Combined valve surgery, n (%)**	101 (20.7)	18 (18)		
**Surgery type III**			2.774	0.096
**Minimally invasive procedures, n (%)**	98 (20.1)	7 (11.3)		
**Full median sternotomy, n (%)**	390 (79.9)	55 (88.7)		
**Operation of LAA, n (%)**	169 (34.6)	25 (40.3)	0.78	0.377
**CABG, n (%)**	17(3.5)	2 (3.2)	0.000	1

CPB, Cardiopulmonary bypass; CABG, Coronary artery bypass grafting; LAA, Left atrial appendage

### Independent risk factors for PMV

Univariate analyses were performed on 550 patients to identify potential risk factors for PMV using baseline characteristics (Table 1), preoperative data (Table 2), and procedure-related data (Table 3). The results indicated that the following 12 factors might be associated with the risk of PMV in patients undergoing valve surgery: age, body mass index (BMI), heart rate (HR), current smoking, left atrial diameter index, NT-proBNP, preoperative white blood cell count (WBC), red blood cell count (RBC), platelet count, intraoperative blood transfusion, cardiopulmonary bypass time, and aortic clamping time. Subsequently, multivariate logistic regression analysis was performed on the aforementioned potential risk factors with a significance level of *P* < 0.05. The analysis revealed that age (OR = 1.082, 95% CI = 1.042–1.125; *P* = 0.000), current smoking (OR = 1.953, 95% CI = 1.007–3.387; *P* = 0.047), left atrial internal diameter index (OR = 1.04, 95% CI = 1.002–1.081; *P* = 0.041), preoperative red blood cell count (OR = 0.049, 95% CI = 0.275–0.876; *P* = 0.016), and aortic clamping time (OR = 0.017, 95% CI = 1.005–1.057; *P* < 0.017) were identified as independent risk factors for the development of PMV. Other characteristics did not demonstrate significant associations (Table 4).

**Table 4:** Multivariate logistic regression analysis of screened variables

Variables	B	S.E.	Wald	*P*-value	OR	95%CI
**Age**	0.079	0.020	16.319	0.000	1.082	(1.042–1.125)
**BMI**	-0.008	0.048	0.031	0.861	0.992	(0.903–1.089)
**HR**	-0.016	0.011	2.28	0.131	0.984	(0.963–1.005)
**Current_smoker**	0.67	0.338	3.928	0.047	1.953	(1.007–3.787)
**LADi**	0.04	0.019	4.175	0.041	1.04	(1.002–1.081)
**NT-proBNP**	0.00	0.000	0.173	0.678	1	(1.000–1.000)
**WBC count**	-0.136	0.114	1.407	0.235	0.873	(0.698–1.093)
**RBC count**	-0.712	0.296	5.8	0.016	0.49	(0.275–0.876)
**Platelet count**	0.001	0.003	0.062	0.804	1.001	(0.995–1.006)
**CPB time**	-0.012	0.01	1.463	0.226	0.988	(0.969–1.007)
**Aortic cross-clamp time**	0.031	0.013	5.684	0.017	1.031	(1.005–1.057)
**Intraoperative blood transfusion**	0.123	0.411	0.089	0.765	1.131	(0.505–2.530)

BMI, body mass index; HR: Heart Rate; LADi, Left atrial diameter index; NT-proBNP, N-terminal pro-brain natriuretic peptide; WBC, white blood cell; RBC, Red blood cell; CPB, Cardiopulmonary bypass.

### Development of predictive models

**Fig. 2:**
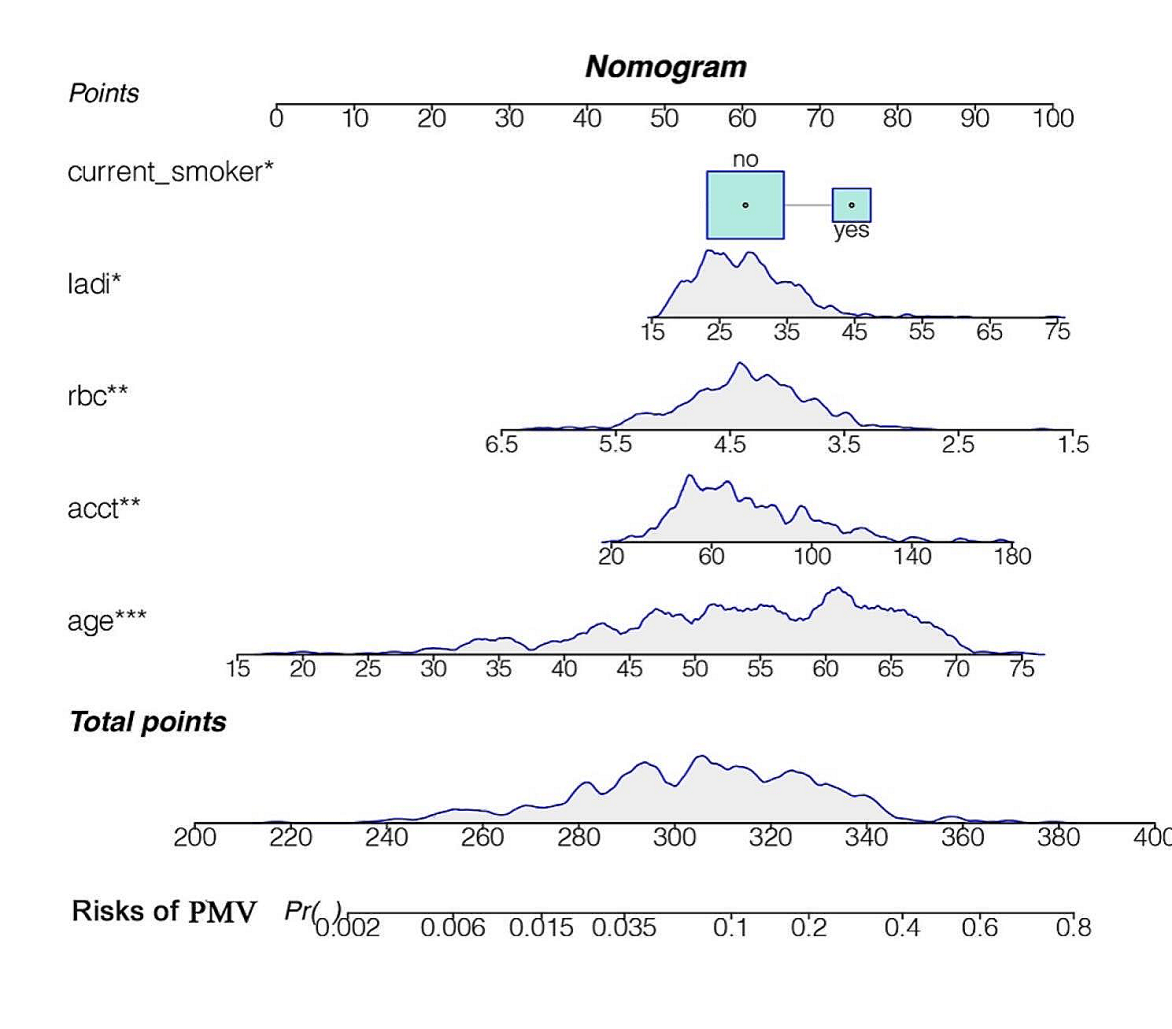
The nomogram for predicting PMV following cardiac valve surgery. ladi, Left atrial diameter index; rbc, red blood cell count; acct, Aortic cross-clamp time

**Fig. 3:**
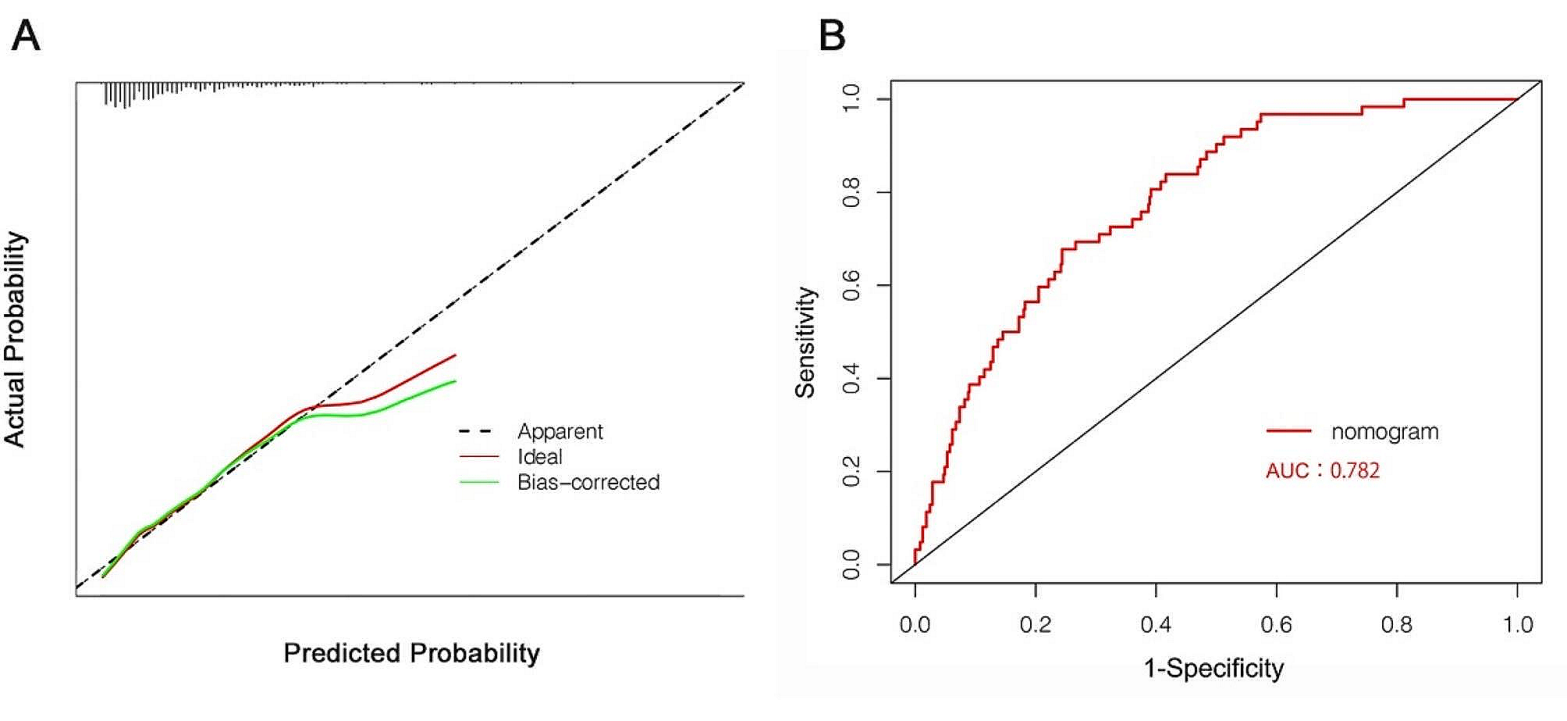
Calibration plots of the nomogram for predicting PMV after valve surgery (**A**) and the ROC curve for the nomogram (**B**)

**Fig. 4:**
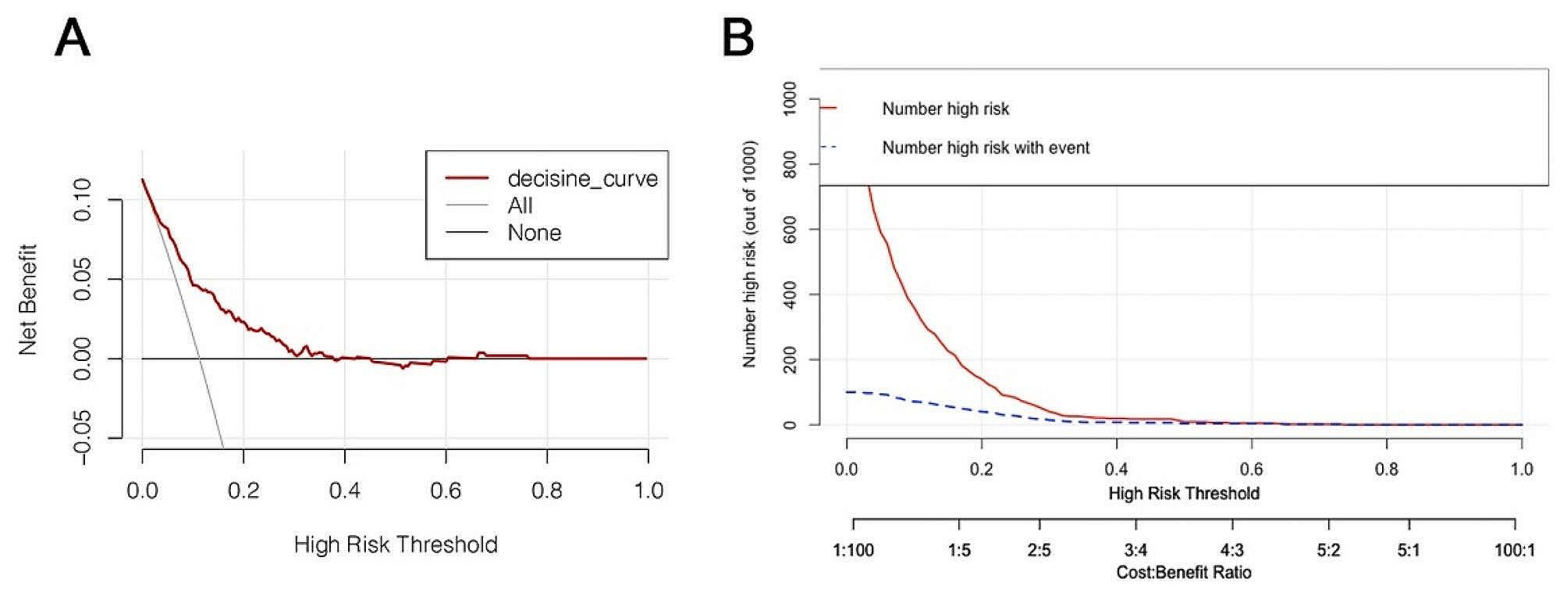
The decision curve analysis curves and the clinical impact curves of the nomogram model. **A**, The decision curve analysis curves. (The horizontal coordinate of the curve is the Threshold Probability, and the vertical coordinate is the Net Benefit Rate. The net benefit rate when everyone is a non-patient and no clinical intervention is represented by the None line, while the net benefit rate when everyone is a patient and clinical intervention is represented by the All line); **B**, The clinical impact curves. (The horizontal coordinates of the clinical impact curves are the probability thresholds, and the vertical coordinates are the number of people. The red curve represents the number of people judged by the model to be at high risk at different probability thresholds, and the blue dashed line represents the number of people judged by the model to be at high risk at different probability thresholds and who have an outcome event. The straight lines below represent the ratio of losses to benefits at different probability thresholds.)

A nomogram was constructed using a multivariate logistic regression model that screened five independent risk factors for PMV. Each variable was assigned a score based on its regression coefficient, as illustrated in Fig. 2. By scoring the variables above and aggregating the scores of all factors in patients, we could forecast the probability of PMV occurrence. Internal validation was conducted through 1000 bootstrap resamples to assess the prediction model’s performance. The model exhibited a C-index of 0.782, as determined by the bootstrap analysis. The X-axis of the calibration curve represents the predicted probability of the model, and its Y-axis represents the actual probability. The diagonal dashed line represents a perfect prediction of the ideal model. The solid line presents the performance of the nomogram, and the closer the two lines fit to the diagonal dashed line, the better the predictive consistency of the nomogram. The calibration curve (Fig. 3A) notably demonstrated good concordance between the predicted and observed outcomes. The ROC curve (Fig. 3B) also displayed an AUC of 0.782 and an accuracy of 0.884. Furthermore, DCA (Fig. 4A) provided insight into the potential clinical benefits of the prediction model, while the clinical impact curve (Fig. 4B) underscored its robust predictive capabilities and clinical utility.

## Discussion

Prolonged mechanical ventilation is a prevalent and significant complication following cardiac surgery. It not only serves as an independent prognostic factor for extended hospital stays, heightened medical expenses, and readmission to the ICU, but also correlates with diminished quality of life, heightened risk of complications, and mortality subsequent to surgery [18, 19]. Incidences of PMV reported in various studies have exhibited a range from 2.6 to 22.7%. The variability in these figures could be attributed to discrepancies in the definition of PMV, which encompassed durations varying from 12 h to 14 days across different studies [19–22]. For the present study, PMV was defined as mechanical ventilation exceeding 24 h after surgery, a time frame recognized by the National Quality Foundation and endorsed by the Society of Thoracic Surgeons (STS) as a quality measure [23]. This study revealed an incidence of PMV to be 11.27%, consistent with findings from other observational studies [21].

Ashwin et al. demonstrated that PMV occurred in approximately 15% of patients undergoing various cardiac surgeries. They identified several factors associated with PMV, including prior cardiac surgery, critical preoperative condition, left ventricular insufficiency, renal insufficiency, and duration and complexity of cardiopulmonary bypass [24]. Shahram et al. indicated that continuous mechanical ventilation for more than 24 h in patients undergoing coronary artery grafting was associated with a history of obstructive lung disease, renal disease, respiratory rate, systolic blood pressure at 24 h postoperatively, creatinine levels at 24 h postoperatively, preoperative ejection fraction, and cardiac enzymes (CK-MB) [25]. While several studies have investigated PMV in the postoperative context, identifying risk factors and developing predictive models for PMV following heart valve surgery remains unexplored. In this study, through univariate and multifactorial analyses, we identified age, current smoking, left atrial diameter index, red blood cell count, and aortic clamping time as independent factors influencing the occurrence of PMV in patients undergoing valve surgery. By combining these variables, a predictive nomogram was successfully constructed. The nomogram is based on the magnitude of the regression coefficients of each independent variable affecting the outcome event, and each variable is assigned a value level. Each level of each factor on the nomogram has a corresponding score, and the total score is obtained by adding up the corresponding scores of each factor. Then, the predicted probability of the onset of PMV can be obtained according to the predictive value corresponding to the total score. The nomogram exhibited good discriminatory power (C-index of 0.782) and demonstrated well-calibrated predictive performance.

This study showed a significant association between age and PMV, corroborating findings from prior research endeavors. Notably, the PMV group in this study exhibited a mean age of 61.5 years, which aligns closely with a previous investigation focusing on mechanically ventilated patients following coronary artery bypass grafting, wherein the PMV group displayed a mean age of 61.9 years [25, 26]. Given that advanced age is often accompanied by a higher likelihood of encountering various ailments, including respiratory and cardiovascular disorders, it is imperative to devote increased attention to providing care for this particular demographic.

In the present investigation, a notable escalation in the occurrence of PMV was observed in individuals identified as current smokers. Zhang et al. substantiated a direct correlation between smoking and the occurrence of PMV after cardiovascular surgical interventions [27]. Smoking confers a heightened vulnerability in patients toward respiratory decompensation and respiratory ailments. Current smokers exhibit an increased susceptibility to PMV owing to diminished lung capacity following surgery and a compromised ability to synchronize with mechanical ventilation [17, 28]. Consequently, such individuals necessitate heightened vigilance and meticulous care from healthcare providers and nurses in the specialized setting of cardiac surgery intensive care units.

This investigation demonstrates the independent association between the left atrial diameter index and PMV risk. Profound cardiac enlargement hampers proper lung expansion and compromises pulmonary function, contributing to extended postoperative ventilation in patients [29, 30]. Concurrently, expanding the left atrial diameter also elevates the likelihood of atrial fibrillation and heart failure [31, 32]. Previous research has established that impaired left ventricular function is linked to postoperative PMV, and atrial fibrillation also serves as a risk factor for PMV [24, 27].

Antonino et al. have demonstrated a significant correlation between preoperative anemia and prolonged postoperative mechanical ventilation (MV) in patients undergoing repeat cardiac surgery, corroborating the present study’s findings [33]. Some clinical studies have suggested that preoperative anemia may be associated with multiple adverse outcomes and increased mortality after cardiac surgery [34, 35]. Patients afflicted with anemia typically exhibit poor physical condition, frailty, or the presence of multiple comorbidities, which may contribute to the protracted requirement for postoperative MV [36]. Consequently, the preoperative correction of anemia in patients assumes paramount importance.

The association between the duration of aortic clamping and the occurrence of postoperative PMV was investigated in this study. In a study conducted by Muhammad et al., it was demonstrated that patients undergoing cardiovascular surgery and experiencing aortic clamping for approximately 80 min were more likely to develop PMV, which aligns with the findings observed in our study [37]. However, in the multifactorial analysis, the duration of cardiopulmonary bypass time did not exhibit a significant association with PMV. This disparity in results could be attributed to variations in surgical procedures and the sample size of the included patients.

There are several limitations inherent in this study. Firstly, the nomogram developed in this research was derived from a single-center, retrospective study, which entailed a relatively small sample size and lacked external validation. Consequently, further investigations are necessary to validate the accuracy and generalizability of this predictive model. Secondly, Since patient information relies mainly on written records, certain important details may have been inadvertently overlooked due to the limited nature of these records, such as the patient’s physical condition, levels of frailty, or nutritional status. Thirdly, due to excessive missing data, certain crucial clinical data could not be included in our study. The median ventilation time of the PMV patients was not too significantly different from that of the patients in the non-PMV group, which may also make our results miss some important factors contributing to the occurrence of PMV. Fourthly, our study solely focused on preoperative and intraoperative data without collecting postoperative information or following up on the patient’s prognosis.

## Conclusion

In conclusion, within our study cohort, the occurrence rate of PMV was 11.27%. To determine potential predictors of PMV, we conducted an extensive examination of five key factors. We successfully established a clinical nomogram, which exhibited commendable performance in discrimination and calibration. The model holds promise for aiding clinicians in identifying individuals at elevated risk of PMV, thereby enabling targeted preventive measures to mitigate this condition.

## Data Availability

The datasets used and analyzed during the current study are available from the corresponding author on reasonable request.
